# Modulation of Root Nitrogen Uptake Mechanisms Mediated by Beneficial Soil Microorganisms

**DOI:** 10.3390/plants14172729

**Published:** 2025-09-02

**Authors:** Francisco Albornoz, Liliana Godoy

**Affiliations:** 1 Departamento de Ciencias Vegetales, Facultad de Agronomía y Sistemas Naturales, Pontificia Universidad Católica de Chile, Santiago 7820436, Chile; 2 Departamento de Fruticultura y Enología, Facultad de Agronomía y Sistemas Naturales, Pontificia Universidad Católica de Chile, Santiago 7820436, Chile; liliana.godoy@uc.cl

**Keywords:** nitrate transporters, ammonium transporters, plant-growth promoting microorganisms, volatile organic compounds

## Abstract

A diverse array of soil microorganisms exhibit plant growth-promoting (PGP) traits, many of which enhance root growth and development. These microorganisms include various taxa of bacteria, fungi, microalgae and yeasts—some of which are currently used in biofertilizers and biostimulant formulations. Recent studies have begun to unravel the complex communication between plant roots and beneficial microorganisms, revealing mechanisms that modulate root nitrogen (N) uptake beyond atmospheric N_2_ fixation pathways. Root N uptake is tightly regulated by plants through multiple mechanisms. These include transcriptional and post-transcriptional control of plasma membrane-localized N transporters in the epidermis, endodermis, and xylem parenchyma. Additionally, N uptake efficiency is influenced by vacuolar N storage, assimilation of inorganic N into organic compounds, and the maintenance of electrochemical gradients across root cell membranes. Many of these processes are modulated by microbial signals. This review synthesizes current knowledge on how soil microorganisms influence root N uptake, with a focus on signaling molecules released by soil beneficial microbes. These signals include phytohormones, volatile organic compounds (VOCs), and various low-molecular-weight organic compounds that affect transporter expression, root architecture, and cellular homeostasis. Special attention is paid to the molecular and physiological pathways through which these microbial signals enhance plant N acquisition and overall nutrient use efficiency.

## 1. Introduction

Nitrogen (N) is an essential macronutrient for plant growth and development and is widely recognized as one of the primary limiting factors for crop productivity [1]. Plants acquire N primarily in the form of ammonium (NH_4_^+^) and nitrate (NO_3_^−^), which are absorbed by root cells through highly regulated physiological and metabolic processes. Uptake initiates at the root surface and involves specific plasma membrane transporters that mediate N translocation across the epidermis and endodermis. Once inside the root, nitrogen-containing compounds follow diverse metabolic and transport pathways, including vacuolar storage, assimilation into organic molecules such as amino acids and nucleotides, or loading into the xylem for translocation to aerial tissues [2]. In certain cases, N may be released back into the rhizosphere via root exudation or efflux mechanisms [2]. The fate of absorbed N is largely governed by the spatial and temporal nutrient demand of different tissues [3]. Consequently, increased N availability generally promotes biomass accumulation and overall plant growth by supporting key biosynthetic and physiological functions.

Beneficial soil microorganisms enhance plant N acquisition through multiple, often synergistic mechanisms. These include increasing N availability in the rhizosphere, stimulating root system development, interacting with root N uptake pathways, and accelerating the assimilation of inorganic N into organic forms within plant tissues [4]. Microbial activity contributes to the mineralization of organic matter, solubilization of bound N compounds, and—particularly in diazotrophs—biological N fixation, thereby enriching the root zone with accessible N sources [5]. At the community level, plant N uptake shapes rhizosphere assembly. Rapid NO_3_^−^ extraction by roots generates C:N gradients that select for bacteria adapted to low-N conditions, reducing overall microbial diversity but enriching functional guilds that further promote N availability [6]. Conversely, targeted management practices—such as breeding for biological nitrification inhibition in sorghum, applying high-C:N amendments or biochar, and tailored inoculations of N_2_ fixers, arbuscular mycorrhizal fungi (AMF), and PGP rhizobacteria—can steer rhizosphere composition toward cooperative consortia that maximize plant-accessible N [7].

Liu et al. [8] employed an in situ isotopic tracing approach by injecting ^15^NH_4_^+^, ^15^NO_3_^−^ and ^15^N-glycine at multiple soil depths to partition N uptake between roots and microorganisms under realistic field conditions. By combining soil fumigation–extraction and isotope ratio mass spectrometry analysis, they revealed spatial and chemical gradients in N use, showing that microbial biomass can outcompete roots for inorganic N pools, although plants and microorganisms exploit distinct niches in space and time. Among rhizobacteria, *Pseudomonas* spp. has garnered attention for its ability to secrete ammonifying enzymes that provide new source of N for root uptake [9,10,11].

Zhu et al. [12] conducted a long-term N addition experiment in temperate broad-leaved forests to investigate how chronic N deposition alters root nutrient-foraging strategies via shifts in rhizosphere fungal communities. Applying 50 kg N ha^−1^ yr^−1^, they measured specific root length and fungal diversity using amplicon-based sequencing in both arbuscular mycorrhizal (AM) and ectomycorrhizal (ECM) tree species. Nitrogen enrichment significantly increased specific root length and overall fungal diversity in both mycorrhizal types, but with contrasting effects: in AM species, N reduced beneficial fungal diversity and increased pathogen diversity, whereas in ECM species, it enhanced beneficial fungi and suppressed pathogens. Moreover, under N addition, pathogen diversity became positively correlated with specific root length, reversing the negative relationship observed under control conditions. Structural equation modeling indicated that indirect N effects on root architecture were mediated through changes in beneficial fungal diversity. These findings highlight how long-term N deposition can reconfigure plant–microbe interactions to modulate root morphology.

Rhizosphere microbial communities form dynamic and functionally diverse consortia that play critical roles not only in nutrient acquisition but also in plant development and adaptation to abiotic and biotic stresses. These microorganisms influence host physiology through both direct and indirect mechanisms. Direct effects include the production of extracellular enzymes (e.g., proteases, ureases, nitrilases) that release nitrogenous compounds from organic substrates. Indirectly, they modulate root architecture, promote root hair formation, and regulate the expression and activity of N transporters such as nitrate transporters (NRTs) and ammonium transporters (AMTs) [13,14].

Beyond nutrient mobilization, rhizosphere microorganisms engage in chemical communication with host plants via a wide array of signaling molecules, including phytohormones (e.g., auxins, cytokinins, gibberellins), volatile organic compounds (VOCs), low-molecular-weight organic acids, and other bioactive metabolites [14,15]. These signals can trigger systemic responses in the host, including transcriptional reprogramming of genes involved in nutrient uptake, stress tolerance, and growth regulation.

At the molecular level, plant–microbe interactions involve the regulation of transcriptional and post-transcriptional networks. Microbial signals have been shown to influence chromatin remodeling, histone modifications, and DNA methylation, thereby modulating the expression of genes associated with N metabolism, root development, and defense responses [16]. This complex interplay reflects a co-evolutionary relationship characterized by metabolic interdependence and adaptive plasticity, enabling plants to optimize resource acquisition under fluctuating environmental conditions.

The present article reviews the current knowledge on the interaction between plants and beneficial soil microorganisms that promote N uptake from the soil. This article is focused on the effect of microorganisms on root growth, the processes of N short- and long-distance transport within the plant, as well as N assimilation and storage. Atmospheric N-fixing microorganisms are not included since they have been comprehensively addressed in recent publications by Sharma et al. [17], Timofeeva et al. [18] and Goyal et al. [19].

## 2. Promotion of Root Growth by PGP Microorganisms

Effective rhizosphere exploration depends on the root system’s ability to grow and extend into a substantial volume of soil. Consequently, any factor that promotes root growth enhances the plant’s ability to absorb N, as a larger root volume increases the probability of encountering N-containing compounds. Root development comprises three distinct zones: the apical meristem, the elongation zone, and the differentiation (or maturation) zone [20]. The apical meristem, located at the root tip, contains undifferentiated cells that divide continuously, pushing older cells forward and driving root elongation. Immediately behind this region lies the elongation zone, where cells expand in size, further extending the root into the soil. The differentiation zone follows, where cells specialize into functional tissues such as xylem, phloem, and root hairs. Two major classes of plant hormones—auxins and cytokinins—regulate root growth. Auxins promote cell elongation and differentiation, whereas cytokinins stimulate cell division and root hair formation [21].

Rhizospheric and endophytic microorganisms have evolved sophisticated mechanisms to biosynthesize phytohormones that mimic or modulate endogenous plant hormonal signaling, thereby influencing plant development and stress responses. Among these, indole-3-acetic acid (IAA), a key auxin, is synthesized by various bacterial species—including *Azospirillum brasilense*, *Pseudomonas fluorescens*, and *Bacillus subtilis*—primarily via the indole-3-pyruvate (IPA) and tryptamine pathways. Recent studies have also identified similar biosynthetic capabilities in several microalgae species [22]. Microbially derived IAA can directly activate plant auxin response factors by de-repressing the Aux/IAA repressor complex [23], leading to the transcription of auxin-responsive genes such as LAX, GH3, and SAUR [24]. This signaling cascade promotes root meristem expansion, rhizodermal cell elongation, and enhanced nutrient foraging capacity.

Elevated IAA levels also stimulate lateral root initiation, as demonstrated in studies involving Pseudomonas and Azospirillum species, resulting in a more extensive root system and increased surface area for nutrient and water uptake [25,26]. Beyond IAA, several PGP microorganisms produce additional phytohormones—including cytokinins, gibberellins, and ethylene modulators such as ACC deaminase—which further influence root architecture and mitigate abiotic stress. For instance, ACC deaminase activity reduces ethylene levels in roots, alleviating stress-induced inhibition of root elongation [27]. The synergistic effects of these microbial metabolites not only enhance root system architecture but also contribute to improved plant resilience and productivity under suboptimal environmental conditions.

Cytokinin production by certain rhizobacteria, such as *Paenibacillus polymyxa*, plays a pivotal role in modulating shoot–root axis development via plant two-component signaling systems [28]. These systems involve histidine kinases (HKs) and downstream response regulators such as ARR1, ARR10, and ARR12, which mediate transcriptional responses to cytokinin perception [29]. Bacterial cytokinins can mimic or amplify endogenous cytokinin signaling, altering the expression of genes involved in cell division, differentiation, and nutrient transport. This modulation reconfigures meristematic activity in both the root and shoot apical meristems, influencing organogenesis and vascular patterning. Under stress or nutrient-deficient conditions, microbial cytokinin signaling contributes to adaptive developmental plasticity by reallocating resources between root and shoot systems. For example, elevated cytokinin levels can suppress primary root elongation while promoting lateral root formation and shoot growth, thereby optimizing nutrient uptake and photosynthetic efficiency [30]. Additionally, cytokinin-mediated cross-talk with other hormonal pathways—including auxin, abscisic acid (ABA), and ethylene—enables fine-tuned regulation of growth responses to environmental cues [31].

Exposure of plant roots to PGP microorganisms can significantly increase endogenous hormone concentrations (Table 1). For instance, inoculation of maize roots with the soil bacteria *Burkholderia phytofirmans* has been reported to increase IAA by 16–22%, an effect further amplified by the addition of L-tryptophan, resulting in a 55% increase in IAA concentration [32]. This rise in IAA levels was significantly and positively correlated with plant N content [33].

The integration of auxin and cytokinin signaling pathways plays a central role in regulating plant developmental responses under variable N availability. These phytohormones exhibit both antagonistic and synergistic interactions that modulate root system architecture, meristem activity, and the expression of nitrogen transporters. A key point of convergence is the dual-affinity nitrate transceptor NRT1.1, which is regulated by auxin at both transcriptional and post-translational levels and influences auxin distribution in the root apex [42]. Under low nitrate conditions, NRT1.1 facilitates basipetal auxin transport away from lateral root primordia, thereby suppressing their emergence. In contrast, high nitrate availability inhibits this transport, resulting in localized auxin accumulation and enhanced lateral root development. Cytokinin modulates this response by antagonizing auxin-induced root proliferation and regulating nitrate-responsive gene networks, including NRT1.1 and other nitrogen transporters [43]. This tripartite signaling integration—auxin, cytokinin, and nitrate—enables plants to dynamically adjust root architecture and nutrient uptake efficiency in response to fluctuating nitrogen conditions.

Recent studies have further highlighted the role of nitrogen metabolites such as nitrate, ammonium, and nitric oxide in modulating auxin biosynthesis via key enzymes including TAA1 and AFB3, adding another layer of complexity to this regulatory network [44]. Elucidating the molecular mechanisms underlying this hormonal cross-talk is essential for improving nitrogen use efficiency and guiding the development of crop genotypes with enhanced responsiveness to nutrient signals.

In parallel, microbial small RNAs (sRNAs) and secondary metabolites have emerged as influential regulators of plant RNA interference (RNAi) pathways, modulating gene expression at the post-transcriptional level. Certain rhizospheric and endophytic microbes, such as *Pseudomonas fluorescens* and *Bacillus subtilis*, produce sRNAs that are internalized by plant cells and incorporated into the RNA-induced silencing complex (RISC) [45]. These sRNAs can target specific plant transcripts for cleavage or translational repression, including genes such as ETR1 (ethylene receptor 1), MPK6 (mitogen-activated protein kinase 6), and NAC domain-containing transcription factors, key regulators of hormone signaling and stress responses [46,47].

Beyond MPK6, other members of the MAPK signaling cascade have also been implicated in nutrient regulation. For example, RAF-type MAPKKKs such as RAF14 and RAF79 exhibit strong transcriptional repression under ammonium-rich conditions in *Chlamydomonas reinhardtii*, suggesting a role in nitrogen assimilation and highlighting the broader relevance of MAPK pathways in nutrient-responsive signaling [48].

In addition to sRNAs, microbial secondary metabolites contribute to RNAi modulation. *Bacillus amyloliquefaciens*, for example, produces cyclic lipopeptides and polyketides that can alter the expression of plant RNAi machinery components [49]. These interactions can induce widespread transcriptomic changes, affecting hormonal balance, defense gene expression, and developmental processes. The phenomenon of cross-kingdom RNA interference, where microbial sRNAs directly regulate plant gene expression, represents a novel and increasingly recognized mechanism of plant–microbe communication with significant implications for plant health and resilience [50].

## 3. Regulation of N Uptake Transporters by PGP Microorganisms

Nitrogen (N) uptake in plant roots is mediated by a variety of transmembrane proteins known as transporters. These transporters are specific to either nitrate or ammonium and are categorized into two main groups based on their affinity and the external concentration at which they function: low-affinity transport systems, which operate under high N concentrations, and high-affinity transport systems, which are active under low N concentrations [51]. Nitrate uptake is carried out by members of the NRT1 (also referred to as NPF) and NRT2 transporter families [51], whereas ammonium uptake is governed by proteins in the AMT family [52].

Several studies have reported the upregulation of genes encoding these transporters following exposure of plant roots to various PGP rhizobacteria. For instance, in a soil-based experiment, *Bacillus subtilis* L1 was shown to enhance the expression of genes encoding for NRT2.1 in arabidopsis, lettuce and wheat [53]. Similarly, inoculation of arabidopsis with *Phyllobacterium brassicacearum* STM196 led to increased expression of *NRT2.5* and *NRT2.6* [54]. Another study demonstrated that co-cultivation of arabidopsis and lettuce with *Pseudomonas nitroreducens* resulted in upregulation of high-affinity nitrate transporters in the NRT2 family, while *P. fluorescens* induced the expression of *amt1.3* and concurrently suppressed certain NRT genes, suggesting a shift in preference from nitrate to ammonium uptake [55].

In soil-based experiments, Calvo et al. [56] demonstrated that inoculation of *Arabidopsis thaliana* with three distinct Bacillus-based PGPRs significantly enhanced shoot and root biomass, chlorophyll content, and nutrient accumulation. Twenty-one days after inoculation, quantitative RT-PCR analysis revealed a substantial upregulation-up to several hundredfold-in the transcript levels of five nitrate transporter genes (*AtNRT1.1*, *AtNRT2.1*, *AtNRT1.2*, *AtNRT2.2*, *AtNRT2.3*) and four ammonium transporter genes (*AtAMT1.1*, *AtAMT1.2*, *AtAMT1.3*, *AtAMT1.5*) compared to untreated controls. These results suggest that PGPR-mediated modulation of nitrogen transporter gene expression plays a key role in enhancing N acquisition and promoting plant growth. Complementary findings were reported by Wang et al. [57], who employed transcriptomic profiling and functional genetics to identify a mycorrhiza-specific nitrate uptake pathway in rice grown in low-N soil. Inoculation with *Rhizophagus irregularis* induced expression of the low-affinity nitrate transporter *OsNPF4.5* exclusively in arbuscule-containing cells, establishing a symbiotic nitrate uptake route that contributed up to 42% of total N derived from NO_3_^−^. Knockdown of *OsNPF4.5* led to a 45% reduction in symbiotic nitrogen uptake and impaired arbuscule development, while heterologous expression in *Xenopus oocytes* confirmed its NO_3_^−^ transport capacity. These findings position *OsNPF4.5* as a central component of a conserved mycorrhizal nitrate uptake mechanism across grass species.

**Figure 1:**
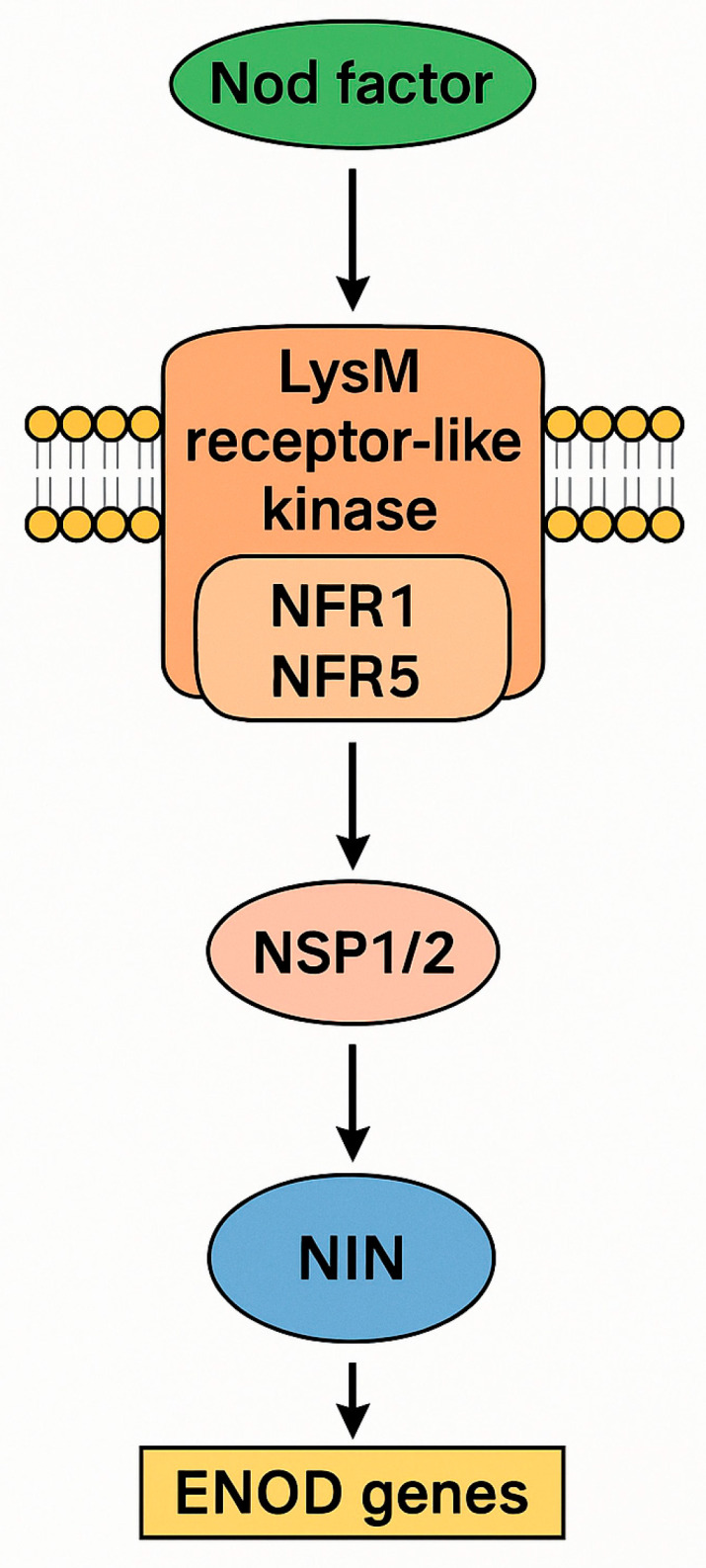
Simplified schematic of the Nod factor-induced signaling pathway in host plants. Bacterial Nod factors are perceived by LysM-type receptors (NFR1/NFR5) located in the plasma membrane, triggering a transcriptional cascade involving NSP1/NSP2. This signaling leads to the activation of NIN and the expression of ENOD genes.

Plant roots exhibit dynamic transcriptomic responses to microbial signals that facilitate nutrient acquisition. In this context, bacteria such as *Stutzerimonas stutzeri* and *Azotobacter vinelandii* possess conserved operons—including *nifHDK*, *nifA*, and *nifB*—whose expression is tightly regulated by environmental factors such as ammonium and oxygen availability [58,59]. In host plants, microbial signals are perceived through LysM-type receptor-like kinases, notably NFR1 and NFR5, which recognize Nod factors and initiate intracellular signaling cascades (Figure 1) [60]. These cascades involve cytoplasmic kinases and transcriptional regulators such as NSP1 and NSP2, which interact with CYCLOPS/IPD3 and DELLA proteins to activate *NIN* [61,62]. Subsequently, *NIN* promotes the transcription of *ENOD* genes, which are essential for nitrogen assimilation [63,64].

Microbial VOCs, such as 2,3-butanediol, acetoin, pentylfuran, isopropyl alcohol, and dimethyl disulfide, serve as airborne signaling molecules capable of modulating plant gene expression at both local and systemic levels [15,65,66]. The role of microbial VOCs in eliciting plant defense responses—particularly induced systemic resistance (ISR) and systemic acquired resistance (SAR)—has been extensively studied. Microbial VOCs from diverse chemical classes, such as organic acids (e.g., butanoic acid), alcohols (e.g., 2-octen-1-ol, 2-octanol. 3-octanol), hydrocarbons (2,4,6-trimethyloctane), ketones (3-methylpentan-2-one, 5-methyl-2-heptanone) and sulfur-containing compounds (dimethyl trisulfide, methanethiol, methyl disulfide), have also been shown to influence the expression of genes involved in nutrient uptake. For instance, tomato plants grown on agar plates and exposed to VOCs emitted by the soil yeast *Solicoccozyma terrea* exhibited increased expression of nitrate transporter genes *NRT1.2*, *NRT2.1*, and *NRT2.3* [15].

The underlying molecular mechanisms often involve VOC-induced reactive oxygen species (ROS) signaling, calcium influx, and activation of mitogen-activated protein kinase (MAPK) cascades, which collectively lead to transcriptional reprogramming in root cells [67,68,69].

Advances in high-throughput sequencing and multi-omics technologies have enabled the development of integrative frameworks that link microbial presence to host transcriptomic, proteomic, and metabolomic reprogramming. For example, transcriptomic analysis of maize roots grown hydroponically and inoculated with *Herbaspirillum seropedicae* revealed upregulation of sugar efflux transporters (SWEET13, SWEET4), malate dehydrogenase, and genes involved in phenylpropanoid biosynthesis, supporting enhanced rhizodeposition and microbial recruitment [70]. Proteomic analyses have identified post-translational modifications of nutrient transporters—such as phosphorylation of *AMT1.1* and ubiquitination of *PHT1;4*—which correlate with distinct stages of microbial colonization. Metabolomic profiling further indicates increased exudation of specialized metabolites, including flavonoids, coumarins, and benzoxazinoids, which act as chemoattractants or signaling molecules for specific microbial taxa [71].

## 4. Regulation of N Assimilation by PGP Microorganisms

Nitrogen assimilation in plants involves a multi-step biochemical pathway beginning with the uptake of nitrate (NO_3_^−^), which is reduced to nitrite (NO_2_^−^) by the enzyme nitrate reductase (NR). Subsequently, nitrite is converted into ammonium (NH_4_^+^) by nitrite reductase (NiR) [72]. The final assimilation steps are catalyzed by glutamine synthetase (GS) and glutamine oxoglutarate aminotransferase (GOGAT), which incorporate NH_4_^+^ into the amino acids glutamine and glutamate [73].

Some PGP rhizobacteria, such as *Bacillus subtilis*, *Azospirillum brasilense*, and *Bradyrhizobium japonicum*, have been shown to enhance NR activity in crops such as lettuce, soybean, and wheat [56,73,74]. This enhancement facilitates the reduction of NO_3_^−^ to NO_2_^−^, a critical step in N assimilation. Notably, *B. japonicum* also increases NiR activity, promoting the subsequent reduction of NO_2_^−^ to NH_4_^+^ [74]. The coordinated upregulation of NR and NiR activities leads to more efficient N assimilation, contributing to improved plant growth and biomass accumulation. These physiological changes are often accompanied by a decrease in tissue NO_3_^−^ levels and an increase in total N content, indicating enhanced N use efficiency (NUE).

Beyond the primary nitrate assimilation pathway, other PGP microorganisms—including *Bradyrhizobium* sp., *Actinomycetes* sp., *Bacillus* sp., and *Paenibacillus graminis*—have been shown to enhance the activity of GS and GOGAT, enzymes responsible for incorporating NH_4_^+^ into amino acids [75]. This pathway is crucial for the synthesis of glutamine and glutamate, which serve as N donors for the biosynthesis of other amino acids and nitrogenous compounds.

Recent advances suggest that plant growth-promoting rhizobacteria (PGPR) influence nitrogen uptake and metabolism not only through hormonal signaling and root architectural changes but also via modulation of the plant epigenome [76]. PGPR have been shown to alter plant microRNA (miRNA) expression profiles, including nutrient-responsive miRNAs, thereby introducing a post-transcriptional regulatory layer that links microbial signaling to nutrient acquisition and environmental adaptation [77]. Epigenetic modifications, particularly histone acetylation (e.g., H3K9ac) and methylation (e.g., H3K27me3), have been implicated in modulating chromatin accessibility and transcriptional activity of metabolic gene clusters, including those involved in vascular development and lignin biosynthesis—processes that indirectly support nutrient transport and assimilation [78,79]. Notably, H3K27me3 has emerged as a key regulator of metabolic gene expression in Arabidopsis thaliana, underscoring the role of chromatin dynamics in plant adaptive responses.

Furthermore, biotic stress and hormonal cues such as salicylic acid can induce locus-specific changes in DNA methylation, coordinating transcriptional responses at critical regulatory loci. Although direct epigenetic regulation of nitrogen assimilation genes such as GS, GOGAT, or NiR has not yet been conclusively demonstrated, accumulating evidence from PGPR-mediated transcriptional and chromatin remodeling suggests that such regulation may contribute to long-term adjustments in plant nutrient metabolism and adaptive capacity [80,81]. This represents a promising avenue for future research.

## 5. Increase in Xylem Transport of N Mediated by PGP Microorganisms

Following N uptake from the soil, a portion is translocated to the aerial parts of the plant via the xylem, where it supports essential physiological processes such as photosynthesis, amino acid biosynthesis, and overall growth. Recent studies have shown that certain PGP rhizobacteria, including *Bacillus amyloliquefaciens* GB03 [82], *Providence* sp. [83], and *Phyllobacterium brassicacearum* STM196 [84], can modulate this translocation process. These microorganisms appear to enhance either the loading of nitrogenous compounds, particularly nitrate (NO_3_^−^), into the xylem or their unloading into leaf tissues, thereby influencing N allocation within the plant.

In *Festuca arundinacea* (tall fescue) grown hydroponically and inoculated with *B. amyloliquefaciens* GB03, a significant increase in shoot N content was observed compared to non-inoculated controls [85]. The authors confirmed the overexpression of two nitrate transporters involved in xylem NO_3_^-^ loading, NRT1.1 and NRT1.5 in agreement with findings reported in *Triticum aestivum* (wheat) and *Arabidopsis thaliana* in response to microbial [83,84].

Importantly, N transport within the plant is not limited to the xylem. Once assimilated into organic forms such as amino acids, N is redistributed via the phloem to actively growing tissues and storage organs. The coordination between xylem and phloem transport is essential for maintaining nitrogen homeostasis and optimizing resource allocation. PGP microorganisms may influence this coordination by modulating the expression of amino acid transporters (e.g., AAPs, LHTs) and altering phloem loading efficiency [85]. Additionally, microbe-induced changes in root architecture and vascular development can enhance the connectivity between xylem and phloem pathways, facilitating more efficient nutrient exchange.

Several PGP microorganisms have been shown to directly influence vascular development. For instance, *Azospirillum brasilense* increased the number and diameter of xylem vessels in maize roots cultivated under soil conditions, potentially improving water and nutrient transport capacity [86]. Similarly, *Pseudomonas fluorescens* has been associated with enhanced vascular differentiation in Arabidopsis, possibly through modulation of auxin signaling pathways [87,88]. *Bacillus subtilis* has also been shown to promote cambial activity and secondary xylem formation in tomato, effects that are likely mediated by changes in cytokinin and gibberellin levels [89]. These structural modifications not only improve the plant’s capacity to transport nutrients but may also contribute to increased resilience under abiotic stress conditions [90].

## 6. Concluding Remarks and Future Perspectives

**Figure 2:**
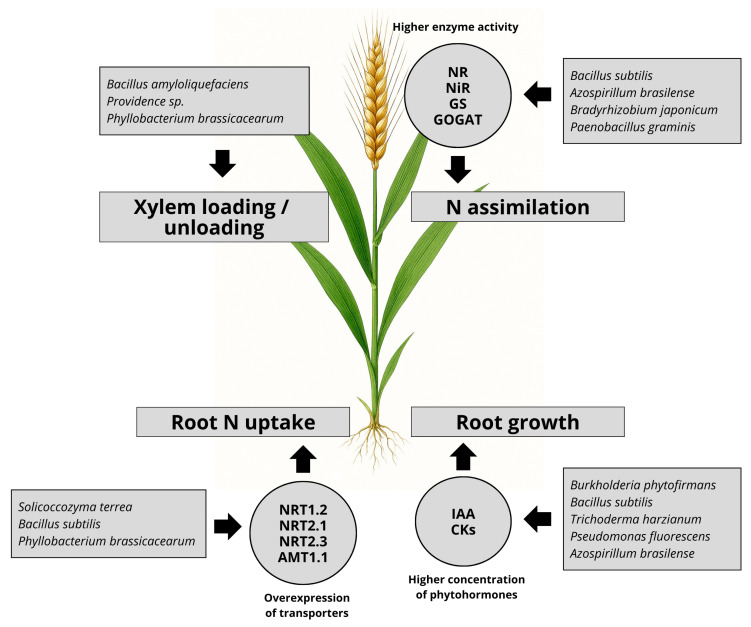
Examples of beneficial microorganisms affecting plant N uptake and metabolism and the mechanisms behind the positive effect. NRT: plasma membrane nitrate transporters; AMT: plasma membrane ammonium transporters; IAA: indole-3-acetic acid; CKs: cytokinins; NR: nitrate reductase; NiR: nitrite reductase; GS: glutamine synthetase: GOGAT: glutamine oxoglutarate amino transferase.

The intricate interactions between plants and beneficial soil microorganisms represent a fundamental component of sustainable nutrient management in agroecosystems. These microorganisms modulate plant physiology processes through the biosynthesis of phytohormones, volatile organic compounds (VOCs), and small RNAs, thereby influencing root system architecture, activating nutrient transporter genes, and eliciting systemic responses (Figure 2). The integration of microbial signals into plant regulatory networks underscores the evolutionary sophistication of plant–microbe symbioses and their critical role in enhancing nitrogen use efficiency (NUE). Specifically, microbial modulation of root nitrogen uptake mechanisms reflects a dynamic equilibrium between competitive and cooperative interactions. Leveraging this interplay—via targeted microbial inoculants, agronomic practices that promote symbiotic associations, and breeding strategies for root traits responsive to microbial cues—offers a promising avenue for improving NUE, reducing dependence on synthetic fertilizers, and advancing the sustainability of cropping systems.

Recent studies have demonstrated that beneficial microorganisms can induce transcriptional and epigenetic reprogramming in plants, particularly in genes associated with nitrogen uptake and metabolism. These changes include the upregulation of nitrate and ammonium transporters (e.g., NRT1.1, NRT2.1, AMT1 family), as well as key enzymes such as nitrate reductase and glutamine synthetase. Epigenetic modifications, including histone acetylation and methylation, have been shown to enhance transcriptional responsiveness and nutrient assimilation. However, the extent to which these molecular alterations translate into agronomically relevant outcomes under field conditions remains variable and context-dependent. Environmental heterogeneity, microbial persistence, soil microbiome competition, and crop genotype interactions often constrain the reproducibility and scalability of these effects. Moreover, the stability and heritability of epigenetic marks across developmental stages and growing seasons are not yet fully characterized, limiting their predictive value for long-term performance. To address these limitations, integrative approaches combining multi-omics analyses, genotype-by-environment–microbiome interaction studies, and long-term field validation are essential. Without such translational frameworks, the agronomic potential of microbially induced molecular enhancements may remain confined to controlled environments, with limited applicability to sustainable agricultural systems. Despite significant progress, several knowledge gaps persist. This includes limiting understanding of the molecular pathways through which microbial metabolites influence plant transcriptional and epigenetic landscapes; designing tailored microbial consortia with complementary functional traits; and the need for long-term field studies to validate the agronomic efficacy of microbial inoculants with respect to crop yield, soil health, and nutrient cycling. This latter point is particularly salient, as most existing studies have been conducted under controlled hydroponic conditions. A comprehensive understanding of the environmental parameters that optimize root zone colonization and the persistence of exogenously applied microorganisms is essential. Moreover, elucidating their interactions with native soil microbiota and assessing potential adverse effects arising from their introduction into the soil environment remains critical research priorities.

Additional areas warranting investigation include the role of beneficial microorganisms in facilitating the uptake of organic N forms, such as amino acids, through the activation of transporters including Amino Acid Transporters (AAT) and Lysine Histidine Transporters (LHT), and their influence on the acquisition and metabolism of micronutrients involved in N assimilation, for example, molybdenum or iron.

In conclusion, leveraging the functional potential of beneficial soil microorganisms offers a promising path toward ecologically sound and resource-efficient agriculture. Continued interdisciplinary research will be pivotal in unlocking the full potential of these microbial allies to meet the growing demands of global food security and environmental sustainability.

## Figures and Tables

**Table 1:** Examples of soil microorganisms with the capacity to synthesize auxins and cytokinins.

Microorganism	Auxin Production	Cytokinin Production	References
*Azospirillum brasilense*	35 µg mL^−1^	30 µg mL^−1^	[34]
*Pseudomonas putida*	20–25 µg mL^−1^	No information available	[35]
*Pseudomonas fluorescens*	10–20 µg mL^−1^	20–30 µg mL^−1^	[36]
*Bacillus subtilis*	5–30 µg mL^−1^	5–25 µg mL^−1^	[37]
*Bacillus amyloliquefaciens*	5–35 µg mL^−1^	10–40 µg mL^−1^	[38]
*Trichoderma harzianum*	20 µg g^−1^ d.w.	0.1–8.3 ng mL^−1^	[39,40]
*Rhizobium* spp.	5–20 µg mL^−1^	5–15 µg mL^−1^	[41]

## Data Availability

No new data were created or analyzed in this study. Data sharing is not applicable to this article.

## Abbreviations

The following abbreviations are used in this manuscript:

GOGAT	Glutamine oxoglutarate amino transferase
GS	Glutamine synthetase
IAA	Indole-3-acetic acid
NiR	Nitrite reductase
NR	Nitrate reductase
PGP	Plant-growth promoting
VOCs	Volatile organic compounds
